# A Simple SQP Algorithm for Constrained Finite Minimax Problems

**DOI:** 10.1155/2014/159754

**Published:** 2014-02-10

**Authors:** Lirong Wang, Zhijun Luo

**Affiliations:** ^1^The Department of Information Science and Engineering, Hunan University of Humanities, Science and Technology, Loudi 417000, China; ^2^The Department of Mathematics and Econometrics, Hunan University of Humanities, Science and Technology, Loudi 417000, China

## Abstract

A simple sequential quadratic programming method is proposed to solve the constrained minimax problem. At each iteration, through introducing an auxiliary variable, the descent direction is given by solving only one quadratic programming. By solving a corresponding quadratic programming, a high-order revised direction is obtained, which can avoid the Maratos effect. Furthermore, under some mild conditions, the global and superlinear convergence of the algorithm is achieved. Finally, some numerical results reported show that the algorithm in this paper is successful.

## 1. Introduction

Consider the following constrained minimax optimization problems:

(1) min⁡x∈Rn F(x),s.t. gj(x)≤0, j∈J={1,2,…,m1},    hl(x)≤0, l∈L={1,2,…,m2},

where *F*(*x*) = max⁡{*f*_*i*_(*x*) | *i* ∈ *I* = {1,2,…, *m*}} and *f*_*i*_(*x*), *g*_*j*_(*x*), *h*_*l*_(*x*) : *R* ^*n*^ → *R* are continuously differentiable.

Minimax problem is one of the most important non-differentiable optimization problems, and it can be widely applied in many fields (such as [1–4]). In real life, a lot of problems can be stated as a minimax problem, such as financial decision making, engineering design, and other fields which wants to obtain the objection functions minimum under conditions of the maximum of the functions. Since the objective function *F*(*x*) is non-differentiable, we cannot use the classical methods for smooth optimization problems directly to solve such constrained optimization problems.

Generally speaking, a lot of the schemes have been proposed for solving minimax problems, by converting the problem (1) to a smooth constrained optimization problem as follows

(2) min⁡ z, s.t. fi(x)≤z, i∈I,     gj(x)≤0, j∈J,     hl(x)=0, l∈L.

Obviously, from the problem (2), the KKT conditions of (1) can be stated as follows:

(3) ∑i∈Iλi∇fi(x)+∑j∈Jμj∇gj(x)+∑j∈Lνj∇hj(x)=0,λi≥0, fi(x)−F(x)≤0, λi(fi(x)−F(x))=0,                        i∈I,μj≥0, gj(x)≤0, μjgj(x)=0, j∈J,∑i∈Iλi=1,  hl(x)=0, l∈L,

where *λ*_*i*_, *μ*_*j*_, and *ν*_*j*_ are the corresponding vector. Based on the equivalent relationship between the K-T point of (2) and the stationary point of (1), a lot of methods focus on finding the K-T point of (1), namely, solving (3). And many algorithms have been proposed to solve minimax problem [5–15]. Such as [5–8], the minimax problems are discussed with nonmonotone line search, which can effectively avoid the Maratos effect. Combining the trust-region methods with the line-search methods and curve-search methods, Wang and Zhang [9] propose a hybrid algorithm for linearly constrained minimax problems. Many other effective algorithms for solving the minimax problems are presented, such as [11–15].

Sequential quadratic programming (SQP) method is one of the efficient algorithms for solving smooth constrained optimization problems because of its fast convergence rate. Thus, it is studied deeply and widely (see, e.g., [16–20], etc.). For typical SQP method, the standard search direction *d* should be obtained by solving the following quadratic programming:

(4) min⁡  ∇F(x)Td+12dTHd, s.t.   gj(x)+∇gj(x)Td≤0, j∈J,       hl(xk)+∇hl(xk)Td=0, l∈L,

where *H* is a symmetric positive definite matrix. Since the objective function *F*(*x*) contains the max operator, it is continuous but non-differentiable even if every constrained function *f*_*i*_(*x*)  (*i* ∈ *I*) is differentiable. Therefore this method may fail to reach an optimum for the minimax problem. In view of this and combining with (2), one considers the following quadratic programming through introducing an auxiliary variable *z*:

(5) min⁡ z+12dTHd, s.t.  fi(x)+∇fi(x)Td≤z, i∈I,      gj(x)+∇gj(x)Td≤0, j∈J,      hl(xk)+∇hl(xk)Td=0, l∈L.

However, it is well known that the solution *d* of (5) may not be a feasible descent direction and can not avoid the Maratos effect. Recently, many researches have extended the popular SQP scheme to the minimax problems (see [21–26], etc.). Jian et al. [22] and Q.-J. Hu and J.-Z. Hu [23] process pivoting operation to generate an *ɛ*-active constraint subset associated with the current iteration point. At each iteration of their proposed algorithm, a main search direction is obtained by solving a reduced quadratic program which always has a solution.

The feasible direction method (MFD) (see [27, 28], etc.) is another effective way for solving smooth constrained optimization problems. An advantage of MFD over the classical SQP method is that a feasible direction of descent can be obtained by solving only one quadratic programming. In this paper, to obtain a feasible direction of descent and reduce the computational cost, we construct a new quadratic programming subproblem. Suppose *x* ^*k*^ is the current iteration point; at each iteration, the descent direction *d* ^*k*^ is obtained by solving the following quadratic programming subproblem:

(6) min⁡ z+12dTHkd, s.t.   fi(xk)+∇fi(xk)Td−F(xk)≤z, i∈I,        gj(xk)+∇gj(xk)Td≤ηkz, j∈J,        hl(xk)+∇hl(xk)Td=0, l∈L,

where *H*_*k*_ is a symmetric positive definite matrix and *η*_*k*_ is nonnegative auxiliary variable. In order to avoid the Maratos effect, a height-order correction direction is computed by the corresponding quadratic programming:

(7) min⁡ z+12(dk+d)THk(dk+d), s.t. fi(xk+dk)+∇fi(xk+dk)Td      −F(xk+dk)≤z, i∈I,      gj(xk+dk)+∇gj(xk+dk)Td≤ηkz, j∈J,      hl(xk+dk)+∇hl(xk+dk)Td=0, l∈L.

Under suitable conditions, the theoretical analysis shows that the convergence of our algorithm can be obtained.

The plan of the paper is as follow. The algorithm is proposed in Section 2. In Section 3, we show that the algorithm is globally convergent, while the superlinear convergence rate is analyzed in Section 4. Finally, some preliminary numerical tests are reported in Section 5.

## 2. Description of the Algorithm

Now we state our algorithm as follows.

**Algorithm 1**

*Step 0*. Given initial point *x* ^0^ ∈ *R* ^*n*^, define a symmetric positive definite matrix *H*_0_ ∈ *R* ^*n*×*n*^. Choose parameters *α* ∈ (0, 1/2), *η*_0_ > 0, and *γ* ∈ (0,1). Set *k* = 0.

*Step 1*. Compute (*d* ^*k*^, *z*_*k*_) by the quadratic problem (6) at *x* ^*k*^. Let (*λ* ^*k*^, *μ* ^*k*^, *ν* ^*k*^) be the corresponding KKT multipliers vector. If *d* ^*k*^ = 0, then STOP.

*Step 2*. Compute $\left({\tilde{d}}^{k},{\tilde{z}}_{k}\right)$ by the quadratic problem (7).

Set $\left({\tilde{\lambda }}^{k},{\tilde{\mu }}^{k},{\tilde{\nu }}^{k}\right)$ as the corresponding KKT multipliers vector. If $||{\tilde{d}}^{k}||>||d^{k}||$, set ${\tilde{d}}^{k}=0$.

*Step 3 (the line search)*. A merit function is defined as follows:

(8) $$\begin{array}{c} w\left(x\right)=F\left(x\right)+r\varphi \left(x\right)+r\underset{l\in L}{\sum }\left|h_{l}\left(x\right)\right|, \end{array}$$

where *φ*(*x*) = max⁡{*g*_*j*_(*x*), *j* ∈ *J*; 0} and *r* is a suitable large positive scalar.

Compute *t*_*k*_, the first number *t* in the sequence {1, 1/2, 1/4, 1/8,…} satisfying

(9) $$\begin{array}{c} w\left(x^{k}+td^{k}+t^{2}{\tilde{d}}^{k}\right)\leq w\left(x^{k}\right)-\alpha t{\left(d^{k}\right)}^{T}H_{k}d^{k}. \end{array}$$

*Step 4 (update)*.   Obtain *H*_*k*+1_ by updating the positive definite matrix *H*_*k*_ using some quasi-Newton formulas. Set $x^{k+1}=x^{k}+td^{k}+t^{2}{\tilde{d}}^{k}$, *η*_*k*+1_ = min⁡{*η*_0_, ||*d* ^*k*^||^*γ*^}. Set *k* : = *k* + 1. Go back to Step 1.

## 3. Global Convergence of the Algorithm

For convenience, we denote

(10) $$\begin{array}{c} I\left(x\right)=\left\{i\in I\;|\;f_{i}\left(x\right)=F\left(x\right)\right\},\\ J\left(x\right)=\left\{j\in J\;|\;g_{j}\left(x\right)=\varphi \left(x\right)\right\}. \end{array}$$

In this section, we analyze the convergence of the algorithm. The following general assumptions are true throughout this paper.

(H  3.1) The functions *f*_*i*_(*x*), *i* ∈ *I*, *g*_*j*_(*x*), *j* ∈ *J*, and *h*_*l*_(*x*), *l* ∈ *L*, are continuously differentiable.
(H  3.2) ∀*x* ∈ *R* ^*n*^; the set of vectors

(11) {(−1∇fi(x)),i∈I(x);(0∇gj(x)),j∈J(x); (0∇hl(x)),l∈L}

 is linearly independent.
(H  3.3) There exist *a*, *b* > 0, such that *a*||*d*||^2^ ≤ *d* ^*T*^ *H*_*k*_ *d* ≤ *b*||*d*||^2^, for all *k* ∈ *R* and *d* ∈ *R* ^*n*^.

**Lemma 2**

Suppose that (H 3.1)–(H 3.3) hold, matrix *H*_*k*_ is symmetric positive definite, and (*d* ^*k*^, *z*_*k*_) is an optimal solution of (6). Then

(1) *z*_*k*_ + (1/2)(*d* ^*k*^)^*T*^ *H*_*k*_ *d* ^*k*^ ≤ 0, *z*_*k*_ ≤ 0,
(2) if *d* ^*k*^ = 0, then *x* ^*k*^ is a K-T point of problem (1).

**Proof**

(1) For (0,0) ∈ *R* ^*n*+1^ is a feasible solution of (6) and *H*_*k*_ is positive definite, one has

(12) $$\begin{array}{c} z_{k}+\frac{1}{2}{\left(d^{k}\right)}^{T}H_{k}d^{k}\leq 0,\;\;z_{k}\leq -\frac{1}{2}{\left(d^{k}\right)}^{T}H_{k}d^{k}\leq 0. \end{array}$$

Further, if *d* ^*k*^ ≠ 0, then *z*_*k*_ < 0.

(2) Firstly, we prove *d* ^*k*^ = 0⇔*z*_*k*_ = 0. If *z*_*k*_ = 0, then (1/2)(*d* ^*k*^)^*T*^ *H*_*k*_ *d* ^*k*^ = (1/2)(*d* ^*k*^)^*T*^ *H*_*k*_ *d* ^*k*^ + *z*_*k*_ ≤ 0. For the positive definite property of *H*_*k*_, it has *d* ^*k*^ = 0. On the other hand, if *d* ^*k*^ = 0, in view of the constraints

(13) $$\begin{array}{c} f_{i}\left(x^{k}\right)+\nabla f_{i}{\left(x^{k}\right)}^{T}d^{k}-F\left(x^{k}\right)\leq z_{k},\;i\in I\left(x^{k}\right), \end{array}$$

we have *z*_*k*_ ≥ 0. Combining *z*_*k*_ ≤ 0, we have *z*_*k*_ = 0.

Secondly, we show that *x* ^*k*^ is a K-T point of problem (1) when *d* ^*k*^ = 0. From the problem (6), the K-T condition at *x* ^*k*^ is defined as follows:

(14) Hkdk+∑i∈Iλik∇fi(xk)+∑j∈Jμjk∇gj(xk)+∑l∈Lνlk∇hl(xk)=0,∑i∈Iλik+ηk∑j∈Jμjk=1,λi≥0,0≤λik⊥(fi(xk)+∇fi(xk)Tdk−F(xk)−zk)≤0,                             i∈I,μj≥0,0≤μjk⊥(gj(xk)+∇gj(xk)Tdk−ηkzk)≤0,                           j∈J,hl(xk)+∇hl(xk)Tdk=0, l∈L.

If *d* ^*k*^ = 0, then *z*_*k*_ = 0, and according to the definition of *η*_*k*_ in Step 4, we have *η*_*k*_ = 0. Furthermore, it holds that

(15) ∑i∈Iλi∇fi(x)+∑j∈Jμj∇gj(x)=0,∑i∈Iλi=1,λi≥0, fi(x)−F(x)≤0, λi(fi(x)−F(x))=0,                        i∈I,μj≥0, gj(x)≤0, μjgj(x)=0, j∈J,hl(xk)=0, l∈L.

That is to see that the results hold.

From Lemma 2, it is obvious, if *d* ^*k*^ ≠ 0, that the line search in Step 3 yields is always completed.

**Lemma 3**

If *d* ^*k*^ ≠ 0 and if *r* satisfies *r* ≥ ||*μ*||_*∞*_ and *r* ≥ ||*ν*||_*∞*_, the line search in Step 3 of the algorithm is well defined.

**Proof**

Firstly, we consider the functions $f_{i}(x+td^{k}+t^{2}\tilde{d})$, *i* ∈ *I*, $g_{j}(x+td+t^{2}\tilde{d})$, *j* ∈ *J*, and $h_{l}\left(x+td+t^{2}\tilde{d}\right)$, *l* ∈ *L*, of the Taylor expansion at *x*. Then, we obtain

(16) $$\begin{array}{c} w\left(x+td+t^{2}\tilde{d}\right)=\tilde{w}\left(x;td\right)+o\left(t\right), \end{array}$$

where

(17) w~(x;td)=t{max⁡i∈I{fi(x)+∇fi(x)Td} +r∑j∈Jmax⁡⁡{gj(x)+∇gj(x)Td,0} +r∑l∈L|hl(x)+∇hl(x)Td|}.

$\tilde{w}(x;d)$ is convex as a function of *d*, and thus we have

(18) $$\begin{array}{c} \tilde{w}\left(x;td\right)-w\left(x\right)\leq t\left\{\tilde{w}\left(x;d\right)-w\left(x\right)\right\},\;\forall t\in \left[0,1\right]. \end{array}$$

From the definition of *w*(*x*), $\tilde{w}(x;d)$ and (1), it is easy to obtain

(19) w~(x;d)−w(x)=max⁡i∈I{fi(x)+∇fi(x)Td}−max⁡i∈Ifi(x)−r∑j∈Jmax⁡⁡{gj(x),0}−r∑l∈L|hl(x)|.

On the other hand, from the first equation of (14) we can get

(20) dTHd+∑i∈Iλi∇fi(x)Td+∑j∈Jμj∇gj(x)Td      +∑l∈Lνl∇hl(x)Td=0.

Let *I*_*f*_(*x*; *d*) = {*i*; *f*_*i*_(*x*)+∇*f*_*i*_(*x*)^*T*^ *d* = max⁡_*l*∈*I*_⁡{*f*_*l*_(*x*)+∇*f*_*l*_(*x*)^*T*^ *d*},  *i* ∈ {1,2,…, *m*}}. Since the third formula of (14) implies

(21) $$\begin{array}{c} \lambda_{i}=0,\;\forall i\in \left\{1,2,\ldots ,m\right\}\setminus I_{f}\left(x;d\right), \end{array}$$

then

(22) dTHd+∑i∈If(x;d)λi∇fi(x)Td     +∑j∈Jμj∇gj(x)Td+∑l∈Lνl∇hl(x)Td=0.

For *i* ∈ *I*_*f*_(*x*; *d*), we get

(23) $$\begin{array}{c} \nabla f_{i}{\left(x\right)}^{T}d=\underset{l\in I}{\max}\left\{f_{l}\left(x\right)+\nabla f_{l}{\left(x\right)}^{T}d\right\}-f_{i}\left(x\right),\\ \underset{i\in I}{\sum }\lambda_{i}=1⟹\underset{i\in I_{f}(x;d)}{\sum }\lambda_{i}=1. \end{array}$$

Thus, (22) implies

(24) max⁡i∈I{fi(x)+∇fi(x)Td}  =∑i∈If(x;d)λifi(x)−dTHd   −∑j∈Jμj∇gj(x)Td−∑l∈Lνl∇hl(x)Td.

Substituting the above equality in (19), we can obtain

(25) w~(x;d)−w(x)  ≤−{dTHd+∑j∈Jμj∇gj(x)Td      +r∑j∈Jmax⁡⁡{gj(x);0}      +∑l∈Lνl∇hl(x)Td+r∑l∈L|hl(x)|}.

It follows from (14) that

(26) w~(x;td)−w(x)  ≤−t{dTHd−∑j∈Jμjgj(x)      +r∑j∈Jmax⁡{gj(x);0}      −∑l∈Lνlhl(x)+r∑l∈L|hl(x)|}.

Considering *r* satisfies *r* ≥ ||*μ*||_*∞*_ and *r* ≥ ||*ν*||_*∞*_, then we have

(27) $$\begin{array}{c} \begin{array}{cc} \tilde{w}\left(x;td\right)-w\left(x\right)\leq -td^{T}Hd<0,\;\forall t\in \left[0,1\right]. & \end{array} \end{array}$$

Then, at *x* ^*k*^, we have

(28) $$\begin{array}{c} w\left(x^{k}+td^{k}+t^{2}{\tilde{d}}^{k}\right)-w\left(x^{k}\right)=\tilde{w}\left(x^{k};td^{k}\right)-w\left(x^{k}\right)+o\left(t\right). \end{array}$$

Since *α* ∈ (0,1/2), for *t* small enough, it holds that

(29) w(xk+tdk+t2d~k)−w(xk)  ≤α(w~(xk;tdk)−w(xk))≤−αtdkTHkdk.

That is, the line search condition (9) is satisfied.

In the following of this section, we will show the global convergence of the algorithm. Since {*d* ^*k*^, *z*_*k*_, *λ* ^*k*^, *μ* ^*k*^} is bounded under all the above-mentioned assumptions, we can assume without loss of generality that there exist an infinite index set *K* and a constant η* such that

(30) xk⟶x∗, Hk⟶H∗, ηk⟶η∗, dk⟶d∗,zk⟶z∗, λk⟶λ∗, μk⟶μ∗, k∈K.

**Theorem 4**

The algorithm either stops at the KKT point *x* ^*k*^ of the problem (1) in finite number of steps or generates an infinite sequence {*x* ^*k*^} any accumulation point x* of which is a KKT point of the problem (1).

**Proof**

The first statement is obvious, the only stopping point being in Step  1. Thus, assume that the algorithm generates an infinite sequence {*x* ^*k*^} and (30) holds. The cases *η*_∗_ = 0 and *η*_∗_ > 0 are considered separately.

*Case A *(*η*_∗_ = 0). By Step 4, there exists an infinite index set *K*_1_⊆*K*, such that *d* ^*k*−1^ → 0, *k* ∈ *K*_1_, while, by Step 3, it holds that

(31) lim⁡k∈K1||xk−xk−1||=lim⁡k∈K1||tk−1dk−1+tk−12d~k−1||≤lim⁡k∈K1(||dk−1||+||d~k−1||)=0.

So, the fact that $x^{k-1}\overset{k\in K_{1}}{\to }x^{*}$ implies that $d^{k-1}\overset{k\in K_{1}}{\to }0$. So, from Lemma 2, it is clear that x* is a K-T point of (1).

*Case B *(*η*_∗_ > 0). Obviously, it only needs to prove that *d* ^*k*^ → 0, *k* ∈ *K*. Suppose by contradiction that d* ≠ 0. Since

(32) $$\begin{array}{c} f_{i}\left(x^{k}\right)+\nabla f_{i}{\left(x^{k}\right)}^{T}d^{k}-F\left(x^{k}\right)\leq z_{k},\;i\in I,\\ g_{j}\left(x^{k}\right)+\nabla g_{j}{\left(x^{k}\right)}^{T}d^{k}\leq \eta_{k}z_{k},\;j\in J,\\ h_{l}\left(x^{k}\right)+\nabla h_{l}{\left(x^{k}\right)}^{T}d=0,\;l\in L, \end{array}$$

in view of *k* ∈ *K*, *k* → *∞*, we have

(33) $$\begin{array}{c} f_{i}\left(x^{*}\right)+\nabla f_{i}{\left(x^{*}\right)}^{T}d^{*}-F\left(x^{*}\right)\leq z_{*},\;i\in I,\\ g_{j}\left(x^{*}\right)+\nabla g_{j}{\left(x^{*}\right)}^{T}d^{*}\leq \eta_{*}z_{*},\;j\in J,\\ h_{l}\left(x^{*}\right)+\nabla h_{l}{\left(x^{*}\right)}^{T}d=0,\;l\in L. \end{array}$$

So, the following corresponding QP subproblem (6) at x*

(34) min⁡(d,z)∈Rn+1 z+12dTH∗d, s.t.     fi(x∗)+∇fi(x∗)Td−F(x∗)≤z,                      i∈I,      gj(x∗)+∇gj(x∗)Td≤η∗z, j∈J,      hl(x∗)+∇hl(x∗)Td=0, l∈L,

has a nonempty feasible set. Moreover, it is not difficult to show that (*z*_∗_, d*) is the unique solution of (34). So, it holds that

(35) $$\begin{array}{c} z_{*}<0,\;\nabla f_{i}{\left(x^{*}\right)}^{T}d^{*}\leq z_{*}<0,\;i\in I\left(x^{*}\right),\\ \nabla g_{j}{\left(x^{*}\right)}^{T}d^{*}\leq \eta_{*}z_{*}<0,\;j\in J\left(x^{*}\right). \end{array}$$

For *x* ^*k*^ → x*, *d* ^*k*^ → d*, *k* ∈ *K*, it is clear, for *k* ∈ *K*, *k* large enough, that

(36) $$\begin{array}{c} \nabla f_{i}{\left(x^{k}\right)}^{T}d^{k}\leq \frac{1}{2}\nabla f_{i}{\left(x^{*}\right)}^{T}d^{*}<0,\;i\in I\left(x^{*}\right),\\ \nabla g_{j}{\left(x^{k}\right)}^{T}d^{k}\leq \frac{1}{2}\nabla g_{j}{\left(x^{*}\right)}^{T}d^{*}<0,\;j\in J\left(x^{*}\right). \end{array}$$

From (36), by imitating the proof of [17, Proposition 3.2], we know that the stepsize *t*_*k*_ obtained by the line search is bounded away from zero on *K*; that is,

(37) $$\begin{array}{c} \begin{array}{cc} t_{k}\geq t_{*}=\inf\left\{t_{k},k\in K\right\}>0. & \end{array} \end{array}$$

In addition, from (9) and Lemma 2, it follows that {*f*(*x* ^*k*^)} is monotonous decreasing. So, considering {*x* ^*k*^}_*K*_ → x* and the hypothesis (H 3.1), one holds that

(38) $$\begin{array}{c} \begin{array}{cc} f_{i}\left(x^{k}\right)⟶f_{i}\left(x^{*}\right),\;k\in K,\;\;i\in I\left(x^{*}\right). & \end{array} \end{array}$$

Hence, from (9) and (36)–(38), we get

(39) 0=lim⁡k∈K(fi(xk+1)−fi(xk))≤lim⁡k∈K αtk∇fi(xk)Tdk≤12αt∗∇fi(x∗)Td∗<0.

It is a contradiction. So, d* = 0. Thereby, according to Lemma 2, x* is a KKT point of problem (1).

## 4. Rate of Convergence

In this section, we show the convergence rate of the algorithm. For this purpose, we add the following some stronger regularity assumptions.

(H 4.1) The functions *f*_*i*_(*x*)  (*i* ∈ *I*), *g*_*j*_(*x*)  (*j* ∈ *J*), and *h*_*l*_(*x*)  (*l* ∈ *L*) are twice continuously differentiable.
(H 4.2) The sequence *x* ^*k*^ generated by the algorithm possesses an accumulation point x*, and *H*_*k*_ → *H*_∗_, *k* → *∞*.
(H  4.3) The second-order sufficiency conditions with strict complementary slackness are satisfied at the KKT point x*; that is, it holds that *λ*_*i*_ > 0, *i* ∈ *I*(x*), *μ*_*j*_ > 0, *j* ∈ *J*(x*), and

(40) $$\begin{array}{c} d^{T}\nabla_{xx}^{2}L\left(x^{*},\lambda^{*},\mu^{*},\nu^{*}\right)d>0,\;0\neq d\in S^{*}, \end{array}$$

    where

(41) ∇xx2L(x∗,λ∗,μ∗,ν∗) =∑i∈Iλi∗∇2fi(x∗)+∑j∈Jμj∗∇2gj(x∗)  +∑l∈Lνl∗∇2hl(x∗) =∑i∈I(x∗)λi∗∇2fi(x∗)+∑j∈J(x∗)μj∗∇2gj(x∗)  +∑l∈Lνl∗∇2hl(x∗),S∗={d∈Rn ∣ ∇fi(x∗)Td=∇fik(x∗)Td,           ∀i∈I(x∗),ik∈I(x∗),   ∇gj(x∗)Td=0,   ∀j∈J(x∗),∇hl(x∗)Td=0,∀i∈I(x∗)}.

According to the all stated assumptions (H 4.1)–(H 4.3) and [21, Theorem 2], we have the following results.

**Lemma 5**

The KKT point x* of problem (1) is isolated.

**Lemma 6**

The entire sequence {*x* ^*k*^} converges to x*; that is, *x* ^*k*^ → x*, *k* → *∞*.

**Proof**

The result of this lemma is similar to the proof of [19, Lemma 4.1].

**Lemma 7**

For *k* large enough, it holds that

(1) *d* ^*k*^ → 0 and  *z*_*k*_ → 0,
(2) *λ* ^*k*^ → λ*, *μ* ^*k*^ → μ*, and *ν* ^*k*^ → ν*.

**Lemma 8**

For *k* large enough, ${\tilde{d}}^{k}$ obtained by Step 2 satisfies

(1) (42) $$\begin{array}{c} ||{\tilde{d}}^{k}||=O\left({||d^{k}||}^{2}\right), \end{array}$$
(2) (43) $$\begin{array}{c} h_{l}\left(x^{k}+d^{k}+{\tilde{d}}^{k}\right)=O\left({||d^{k}||}^{3}\right),\;\forall l\in L,\\ g_{j}\left(x^{k}+d^{k}+{\tilde{d}}^{k}\right)=O\left({||d^{k}||}^{3}\right),\;\forall j\in J\left(x\right). \end{array}$$

**Proof**

(1) The result can be proven similarly to the proof of [5, Proposition 3.1] or [19, Lemma 4.3].

(2) We have

(44) hl(xk+dk+d~k) =hl(xk+dk)+∇hl(xk+dk)Td~k+O(||d~k||2) =hl(xk+dk)+(∇hl(xk)+O||dk||)Td~k+O(||d~k||2) =hl(xk+dk)+∇hl(xk)Td~k+O(||dk||3) =O(||dk||3), l∈L.

Analogously, the other result is not difficult to be shown.

To get the superlinearly convergent rate of the above proposed algorithm, the following additional assumption is necessary.

(H 4.4) The matrix sequence *H*_*k*_ satisfies that

(45) $$\begin{array}{c} ||P_{k}\left(H_{k}-\nabla_{xx}^{2}L\left(x^{k},\lambda^{k},\mu^{k},\nu^{k}\right)\right)d^{k}||=o\left(||d^{k}||\right), \end{array}$$

where

(46) Pk=In−Ak(AkTAk)−1AkT,Ak=Ak(xk)=((∇fi(xk)−∇fik(xk)),            ∇gj(xk),∇hl(xk)),(i∈I(xk)∖{ik},j∈J(xk),l∈L(xk)).

According to Lemmas 6 and 8, it is easy to know

(47) ||Pk(Hk−∇xx2L(xk,λk,μk,νk))dk|| =o(||dk||)⟺||Pk(Hk−∇xx2L(x∗,λ∗,μ∗,ν∗))dk|| =o(||dk||).

**Lemma 9**

For *k* large enough, under the above-mentioned assumptions, *t*_*k*_ ≡ 1.

**Proof**

It is only necessary to prove that

(48) $$\begin{array}{c} w\left(x^{k}+d^{k}+{\tilde{d}}^{k}\right)\leq w\left(x^{k}\right)-\alpha d^{kT}H_{k}d^{k}. \end{array}$$

From (6) and (14), we have

(49) fi(xk+dk)=fi(xk)+∇fi(xk)Tdk+O(||dk||2)=f(xk)+zk+O(||dk||2), i∈I(X∗),fj(xk+dk)=fj(xk)+∇fj(xk)Tdk+O(||dk||2)=f(xk)+zk+O(||dk||2), j∈I(X∗).

Hence,

(50) $$\begin{array}{c} f_{i}\left(x^{k}+d^{k}\right)=f_{j}\left(x^{k}+d^{k}\right)+O\left({||d^{k}||}^{2}\right),\;\forall i,j\in I\left(x^{*}\right). \end{array}$$

Similarly, together with $||{\tilde{d}}^{k}||=O({||d^{k}||}^{2})$, it is easy to get

(51) fi(xk+dk+d~k)  =fj(xk+dk+d~k)+O(||dk||3), ∀i,j∈I(x∗).

On the other hand, the facts that *d* ^*k*^ → 0 and $\;\;{\tilde{d}}^{k}\to 0$ imply that $I(x^{k}+d^{k}+{\tilde{d}}^{k})\subseteq I(x^{*})$ (*k* large enough). Thus, for $j_{k}\in I(x^{k}+d^{k}+{\tilde{d}}^{k})\subseteq I(x^{*})$, we have

(52) F(xk+dk+d~k)=max⁡l∈I{fl(xk+dk+d~k)}=fjk(xk+dk+d~k)=fj(xk+dk+d~k)+O(||dk||3), ∀j∈I(x∗).

By the definition of *w*(*x*) and Lemma 8, we have

(53) $$\begin{array}{c} w\left(x^{k}+d^{k}+{\tilde{d}}^{k}\right)=\underset{l\in I}{\max}\left\{f_{l}\left(x^{k}+d^{k}+{\tilde{d}}^{k}\right)\right\}+O\left({||d^{k}||}^{3}\right). \end{array}$$

Multiplying both sides of (52) by *λ*_*i*_^*k*^ and adding them, combining ∑_*l*∈*I*(x*)_ *λ*_*i*_^*k*^ = 1 with (53), we get

(54) $$\begin{array}{c} w\left(x^{k}+d^{k}+{\tilde{d}}^{k}\right)=\underset{i\in I\left(x^{*}\right)}{\max}\left\{f_{i}\left(x^{k}+d^{k}+{\tilde{d}}^{k}\right)\right\}+O\left({||d^{k}||}^{3}\right). \end{array}$$

In addition, for *k* large enough, we have

(55) L(xk+dk+d~k,λik,μjk,νlk)  =∑i∈Iλikfi(xk+dk+d~k)   +∑j∈Jμjkgj(xk+dk+d~k)   +∑l∈Lνlkhl(xk+dk+d~k)  =∑i∈I(x∗)λikfi(xk+dk+d~k)   +∑j∈Jg(x∗)μjkgj(xk+dk+d~k)   +∑l∈Lνlkhl(xk+dk+d~k)  =∑i∈I(x∗)λikfi(xk+dk+d~k)+O(||dk||3).

Combining the above equation with (54) we can obtain

(56) w(xk+dk+d~k) =L(xk+dk+d~k,λik,μjk,νlk)+O(||dk||3) =L(xk,λik,μjk,νlk)+∇xL(xk,λik,μjk,νlk)T(dk+d~k)  +12(dk+d~k)T∇xx2L(xk,λik,μjk,νlk)  ×(dk+d~k)+o(||dk||2) =L(xk,λik,μjk,νlk)+∇xL(xk,λik,μjk,νlk)T(dk+d~k)  +12(dk)T∇xx2L(xk,λik,μjk,νlk)(dk)+o(||dk||2).

From the KKT condition (14) implies ∇_*x*_ *L*(*x* ^*k*^, *λ*_*i*_^*k*^, *μ*_*j*_^*k*^, *ν*_*l*_^*k*^) = −*H*_*k*_ *d* ^*k*^; then we get

(57) ∇xL(xk,λik,μjk,νlk)T(dk+d~k)  =−dkTHkdk+o(||dk||2),  ∑i∈I(x∗)λik=1.

Thus,

(58) w(xk+dk+d~k)  =L(xk,λik,μjk,νlk)−dkTHkdk   +12(dk)T∇xx2L(xk,λik,μjk,νlk)   ×(dk)+o(||dk||2)  =w(xk)−αdkTHkdk+12(dk)T   ×(∇xx2L(xk,λik,μjk,νlk)−Hk)(dk)   −12dkTHkdk+αdkTHkdk   +L(xk,λik,μjk,νlk)−w(xk)+o(||dk||2)  ≤w(xk)−αdkTHkdk   −(12−α)dkTHkdk+o(||dk||2).

For *k* large enough, according to *α* ∈ (0, 1/2), it holds that

(59) $$\begin{array}{c} w\left(x^{k}+d^{k}+{\tilde{d}}^{k}\right)\leq w\left(x^{k}\right)-\alpha d^{kT}H_{k}d^{k}. \end{array}$$

From Lemma 9 and the method of [29, Theorem 5.2], we can get the following.

**Theorem 10**

Under all stated assumptions, the algorithm is superlinearly convergent; that is, the sequence {*x* ^*k*^} generated by the algorithm satisfies ||*x* ^*k*+1^ − x*|| = *o*(||*x* ^*k*^ − x*||).

## 5. Numerical Experiments

In this section, we select several problems to show the efficiency of the algorithm in Section 2. Some preliminary numerical experiments are tested on an Intel(R) Celeron(R) CPU 2.40 GHz computer. The code of the proposed algorithm is written by using MATLAB 7.0 and utilized the optimization toolbox to solve the quadratic programmings (6) and (7). The results show that the proposed algorithm is efficient.

During the numerical experiments, are chosen at random some parameters as follows: *α* = 0.25, *η*_0_ = 1, *γ* = 0.5, and *H*_0_ = *I*, the *n* × *n* unit matrix. *H*_*k*_ is updated by the BFGS formula [16]. In the implementation, the stopping criterion of Step 1 is changed to If ||*d*_0_^*k*^|| ≤ 10^−6^, STOP.

This algorithm has been tested on some problems from [10, 11, 26]. The results are summarized in Table 1. The columns of this table have the following meanings:

  Number: the number of the test problem in [10, 11] or [26];
  
*n*: the dimension of the problem;
  
*m*: the number of objective functions;
  
*m*_1_: the number of inequality constraints;
  
*m*_2_: the number of equality constraints;
  NT: the number of iterations;
  IP: the initial point;
  FV: the final value of the objective function.

## 6. Concluding Remarks

In this paper, we propose a simple feasible sequential quadratic programming algorithm for inequality constrained minimax problems. With the help of the technique of method of feasible direction, at each iteration, a main search direction is obtained by solving only one reduced quadratic programming subproblem. Then, a correction direction is yielded by solving another quadratic programming to avoid Maratos effect and guarantee the superlinear convergence under mild conditions. The preliminary numerical results also show that the proposed algorithm is effective.

As further work, we can get the main search direction by other techniques, for example, sequential systems of linear equations technique. And we can also consider removing the strict complementarity.

## Figures and Tables

**Table 1:** Numerical results of Algorithm 1.

Number	*n*, *m*, *m*_1_, *m*_2_	NT	IP	FV
1 ([10, Problem 1])	2, 3, 0, 0	11	(1, 5)^*T*^	1.952224
2 ([10, Problem 4])	2, 3, 0, 0	10	(3, 1)^*T*^	0.616234
3 ([11, Problem 1])	2, 3, 2, 0	7	(0, 0)^*T*^	1.952224
4 ([11, Problem 2])	2, 6, 2, 0	12	(1, 3)^*T*^	0.616432
5 ([11, Problem 4])	2, 3, 2, 0	10	(4, 2)^*T*^	2.250000
6 ([11, Problem 5])	4, 4, 3, 0	32	(0, 1, 1, 0)^*T*^	−44.000000
7 ([11, Problem 6])	2, 3, 2, 0	4	(0, 1)^*T*^	2.000000
8 ([26, Problem 5])	2, 2, 0, 1	12	(0, 4)^*T*^	−5.875407
9 ([26, Problem 6])	3, 3, 1, 2	5	(2, 3, 2)^*T*^	−3.934502
10 ([26, Problem 7])	10, 8, 0, 3	54	(0, 1, 1, 1, 0, 0, 0, 1, 1, 1, 0)^*T*^	2.3339*e* + 003
